# Whole genome population genetics analysis of Sudanese goats identifies regions harboring genes associated with major traits

**DOI:** 10.1186/s12863-017-0553-z

**Published:** 2017-10-23

**Authors:** Siham A. Rahmatalla, Danny Arends, Monika Reissmann, Ammar Said Ahmed, Klaus Wimmers, Henry Reyer, Gudrun A. Brockmann

**Affiliations:** 1 Albrecht Daniel Thaer-Institut für Agrar- und Gartenbauwissenschaften, Humboldt-Universität zu Berlin, Invalidenstraße 42, D-10115 Berlin, Germany; 2 Department of Dairy Production, Faculty of Animal Production, University of Khartoum, P.O. Box 32, 13314 Khartoum North, Shambat Sudan; 3 Leibniz-Institut für Nutztierbiologie (FBN), Institut für Genombiologie, Wilhelm-Stahl-Allee 2, 18196, Dummerstorf, Germany

**Keywords:** SNP analysis, Genetic diversity, Adaptation, Desert goat, Nilotic goat, Nubian goat, Taggar goat

## Abstract

**Background:**

Sudan is endowed with a variety of indigenous goat breeds which are used for meat and milk production and which are well adapted to the local environment. The aim of the present study was to determine the genetic diversity and relationship within and between the four main Sudanese breeds of Nubian, Desert, Taggar and Nilotic goats. Using the 50 K SNP chip, 24 animals of each breed were genotyped.

**Results:**

More than 96% of high quality SNPs were polymorphic with an average minor allele frequency of 0.3. In all breeds, no significant difference between observed (0.4) and expected (0.4) heterozygosity was found and the inbreeding coefficients (F_IS_) did not differ from zero. F_st_ coefficients for the genetic distance between breeds also did not significantly deviate from zero. In addition, the analysis of molecular variance revealed that 93% of the total variance in the examined population can be explained by differences among individuals, while only 7% result from differences between the breeds. These findings provide evidence for high genetic diversity and little inbreeding within breeds on one hand, and low diversity between breeds on the other hand. Further examinations using Nei’s genetic distance and STRUCTURE analysis clustered Taggar goats distinct from the other breeds. In a principal component (PC) analysis, PC1 could separate Taggar, Nilotic and a mix of Nubian and Desert goats into three groups. The SNPs that contributed strongly to PC1 showed high F_st_ values in Taggar goat versus the other goat breeds. PCA allowed us to identify target genomic regions which contain genes known to influence growth, development, bone formation and the immune system.

**Conclusions:**

The information on the genetic variability and diversity in this study confirmed that Taggar goat is genetically different from the other goat breeds in Sudan. The SNPs identified by the first principal components show high F_st_ values in Taggar goat and allowed to identify candidate genes which can be used in the development of breed selection programs to improve local breeds and find genetic factors contributing to the adaptation to harsh environments.

**Electronic supplementary material:**

The online version of this article (10.1186/s12863-017-0553-z) contains supplementary material, which is available to authorized users.

## Background

Compared to other African countries, goats in Sudan constitute a large part of the livestock population. With an estimated number of 31 million goats (out of 365 million in whole Africa) that produced about 1.532 million tons of milk in 2013, Sudan was the largest producer of goat milk in Africa and the third largest producer in the world [1]. Goats in Sudan have an important contribution to food security by producing milk and meat. Beyond that, manure and skins provide a source of income for farmers. Thus, goats constitute an important source of livelihood, social security and rural economy. Therefore, the improvement of the productivity of local breeds contributes to rural development. Even under the harsh environments, selection of favorable genome variants could sustainably improve productivity. Systematic genetic diversity studies of indigenous adapted breeds would be necessary to understand the acquired unique features of these breeds.

In Sudan, different indigenous goat populations are distributed across all agro-ecological zones from the arid region in the North to the fertile Savannah in the South. The adaptation to the harsh climate and the limited feed resources has led to natural selection of goats for minimal maintenance and low water requirements [2]. They developed a high water economy utilizing their body fluid more effectively which ensures the maintenance of an appropriate dry matter intake during periods of water scarcity and thereby, nonetheless, an adequate level of productivity [3].

Sudanese goats are classified into Nubian, Desert, Nilotic and Taggar goats [4]. The main classifications of these goats can be shortly summarized as follows: Nubian, a normal size dairy goat commonly found in the semi-arid areas in Sudan. Desert is a dual purpose goat kept by the nomadic tribes of Sudan, and is commonly found in the desert areas of Sudan. The Nilotic goat is mostly used for meat production, after Sudan was split into North and South, it is mostly found in the border area between North and South Sudan. Taggar goats are a species of dwarf goats commonly found in mountainous areas all over Sudan.

The aim of this study was to investigate the genetic diversity and population structure of the four above mentioned important indigenous Sudanese goat breeds living in an arid climate and suffering harsh feeding conditions. For the genetic analyses, we used the 50 K goat SNP chip (Illumina, San Diego, CA). The chip provides markers across the whole genome, allowing for precise identification of the whole genome diversity within and among the different populations. Furthermore, the whole genome data made it feasible to identify genomic regions that differ between the breeds and contain genes likely linked to the typical traits of the Sudanese goat breeds.

## Methods

### Animals and sampling

Blood samples were collected for genotyping from each of the four goat breeds, Nubian (NU), Desert (D), Taggar (T), and Nilotic (NI), 24 representative female animals were sampled from different regions of Sudan (Additional file 1: Table S1).

Nubian goats were collected at six locations in four different states along the river Nile, the Dongola area (Northern State), Abu Hamad, Aldamir, Shendi (River Nile State), Khartoum (Khartoum State) and Aljazirah (Aljazirah State). In total Nubian samples were obtained from 10 villages, three districts, one university farm, and three research stations.

Desert goat samples were collected from Bara and Abu Zabad area in the North Kordofan state (eight villages, one university farm).

Taggar goat samples were obtained from the Nuba Mountains and Dalang area in the South Kordofan state (five villages, one research station).

Nilotic goat samples were collected in the Kosti and Rabak areas in the White Nile state (eight villages).

Livestock experts, herdsmen and owners of the animals were consulted to ensure representative sampling of unrelated animals.

The characterization of the different Sudanese goat breeds are the following:

#### Nubian goat

Nubian is a highly productive dairy goat compared to other Sudanese goat breeds. Nubian goats are widely distributed in arid and extreme arid areas [4]. Besides Sudan, Nubian goats are found widespread in North Eastern Africa and the Mediterranean coastal belt. They have likely their origin in Sudan [5]. These goats are commonly black, but pure brown and multi-colorations of black and white also exist [6, 7].

#### Desert goat

Sudanese Desert goats are dual purpose goats. They are mainly found in semi-arid areas of the West of Sudan. They also move to extreme arid areas during nomadic migration. The efficient transformation of low quality feed into body mass makes the Desert goat valuable for the production of meat [8]. Desert goats are phenotypically similar to West African long-legged goats, and are possibly related to the Nubian goats [4]. Coat color is variable and mixed colors exist [9, 10].

#### Taggar goat

Taggar is a meat-type goat that has adapted to survive under harsh environmental conditions [11–13]. It is kept in many parts of Sudan with the highest density in the Nuba Mountain area close to the border to South Sudan. Taggar is a dwarf goat with disproportionally short legs, plump body and short head. The short stature is thought to result from achondroplastic dwarfism with lack of ossification at the cartilage joints [13, 14]. It is assumed that natural selection for the recessive dwarfism gene was favorable in response to the humid and hot climate conditions [4]. The most common coat colors for Taggar goats are dark or grey brown [7, 12].

#### Nilotic goat

Nilotic goat is another meat-type goat that produces high muscle mass under good feeding conditions [15]. Nilotic goats have a high reproductive potential since they reach sexual maturity at an early age. Nilotic goats live in the border region between Sudan and South Sudan. They distinguish from other breeds through their resistance against *Trypanosomiasis* [16]. These goats are small in size; the body is compact, but has normal proportions [6, 7, 17, 18]. Though, different from Taggar goats, achondroplasia does not occur in this breed. Almost all colors occur, but the predominant color is a mixture of black and white.

### Genotyping

Blood samples were collected from the jugular vein using vacutainer tubes containing EDTA as anticoagulant. Blood was stored at −20 °C until DNA was extracted using the Puregen core kit A (Qiagen Sciences, Maryland, USA). All animals were genotyped with the Goat SNP52 BeadChip (Illumina, San Diego, CA), developed by the International Goat Genome Consortium (IGGC) [19]. The raw signal intensities of the 53,347 SNPs on the chip were imaged using the IlluminaScan Reader and converted into genotype calls with GenomeStudio software suite (version 2011.1) by using the SNP genomic locations and cluster file made available by IGGC (ftp://ftp.ncbi.nlm.nih.gov/snp/organisms/goat_9925/viewBatch/snpBatch_IGGC_1057128.gz). Locations of the SNP probes were remapped using BLASTN towards the CHIR 1.0, CHIR 2.0 and LWT01 genome version of *Capra hircus*. This was done to provide future researchers with an easy way to compare our results with their own work, since other researchers can find the corresponding locations on the genome version of their choice. Locations reported in this paper are based on the CHIR1.0 genome build. Locations of protein coding genes on the CHIR1.0 genome were obtained from The National Center for Biotechnology Information (NCBI) [http://www.ncbi.nlm.nih.gov/]; the version used is available as supplemental file (Additional file 2: locations of Protein coding genes).

### SNP quality control

The R language for statistical computing v3.2.3 was used for quality control of the called SNP data [20]. From the entire set of 53,347 SNPs, only those with an Illumina GenTrain score ≥ 0.6 were kept (1441 SNPs failed). Furthermore, all SNPs with a minor allele frequency (MAF) lower than 5% (2273 SNPs) as well as SNPs which showed more than 5% missing data (150 SNPs) were excluded from further analysis. The deviation from Hardy-Weinberg equilibrium (HWE) was calculated and we found only 42 SNPs not in HWE. Since HWE deviations only concerns a very limited amount of SNPs <0.1% of the total, we decided to not remove these SNPs from further analysis. SNP probe sequences were mapped against the CHIR1.0 genome assembly using the Basic Local Alignment Search Tool (BLAST) [21, 22]. Probe sequences that were not mapping in the CHIR1.0 genome (41 SNPs) or probes which showed multiple hits (937 SNPs) against the reference genome were dropped from further analysis. 48,505 SNPs passed the quality control. Based on quality control of the data, one sample (Nubian goat) was excluded from further analysis.

### Statistical analyses

#### Genetic diversity assessment

In order to assess the genetic diversity, the minor allele frequency, distribution and proportion of polymorphic SNPs were computed using the R language for statistical computing v3.2.3 [20]. To measure the genetic variation within a population, mean observed heterozygosity (H_o_) and expected heterozygosity (H_E_) for each breed of goats and the total population were analyzed using the diveRsity R package v.1.9.89 [23]. The average population inbreeding coefficient (F_IS_) using Sewall Wright’s method [24] and the pairwise genetic differentiation between populations (F_st_) were also calculated using the diveRsity package. To detect the level of genetic variation among samples within populations and among populations at different hierarchical levels, the Analysis of MOlecular VAriance (AMOVA) [25] was performed using StAMPP R package [26]. StAMPP calculates an AMOVA based on the Nei’s genetic distance matrix using the amova() function from the package PEGAS for exploring within and between population variation. StAMPP uses the formula: distance = populations, to calculate a hierarchical AMOVA as described in Excoffier et al. [25] to explore population differentiation and within/between population variation.

#### Hierarchical clustering

To measure the genetic distance between the four goat populations, hierarchical clustering of SNP data was performed. Pairwise Nei’s genetic distances [27] between all individuals were calculated from the SNP data by using the StAMPP R package [26]. Additionally, we used Reynolds [28] and Manhattan [29] distances between individuals (Additional file 3: Figure S1). Distances were clustered using the hclust function in R. After clustering of genetic distances between individuals, the ape package [30] was used to convert the resulting dendrogram into a phylogentic tree. For visualization of the phylogenetic tree a standard ape plotting functions was used.

#### STRUCTURE analysis

Population structure was determined by using a model-based clustering for assigning individuals from multi locus genotypes to a population using the STRUCTURE 2.3.3 software suite [31–33]. STRUCTURE uses a maximum likelihood method to infer the genetic ancestry of each individual from a mixture of K pre-defined ancestral groups. STRUCTURE analysis was carried out using an admixture model and correlated allele frequencies. Under the hypothesis of two to five sub-populations K was set from 2 to 5 and the length of the burn-in period was set to 100,000 iterations, followed by 100,000 Markov Chain Monte Carlo (MCMC) iterations. The whole analysis was then repeated five times in STRUCTURE to prevent the model from producing local optimal results. In our analysis, all five runs gave similar results. Results from STRUCTURE were loaded back into the R environment to visualize resulting groups. The most likely hierarchical level of genetic structure was determined by log probability of the data (Ln Pr (X|K) [33] and observed variance of this probability.

#### Principal component analysis (PCA)

As a different approach to characterize divergence, the genetic relationship between animals was analyzed by principal component analysis (PCA) using the ‘prcomp’ function in the R language. PCA is a statistical method for exploring and interpretation of big data by reducing the dimension of the data to the few principal components which capture the majority of the genetic variation observed in the genotypes. We visualized the contribution of individuals to the first 10 principal components; from our data we observe that a combination of PC1 and PC2 shows a good separation between different goat breeds. As such we investigate which SNPs allow PCA to separate between the four populations of Sudanese goats; we investigated which SNPs contribute highly to PC1.

To select high impact SNPs we calculated the variable correlations with PC1 by multiplying the loading factors with the component standard deviations. Quality of representation for all variables on the factor map (cos^2^) was calculated as the squared variable correlation. Afterwards, we summed up all the cos^2^ values for PC1 and expressed each SNP contribution as percentage of total variation. We then looked for SNPs which have more than 10 times the expected contribution to PC1 (contribution ≥0.02%, expected 0.0021%). These SNPs will capture most of the differentiation among breeds separated by PC1. We extracted genes within a region of 500 kb cis-window around these high contributing SNPs according to SNP Annotation and Proxy Search (SNAP) [34].

## Results

The main result from this study is that Taggar goats show significant genetic differences from the other three goat breeds. In all our analysis e.g. F_st_, STUCTURE, clustering and PCA we observe that Taggar goats show significant genetic differences compared to the other breeds.

### Genetic diversity within breeds

**Fig. 1:**
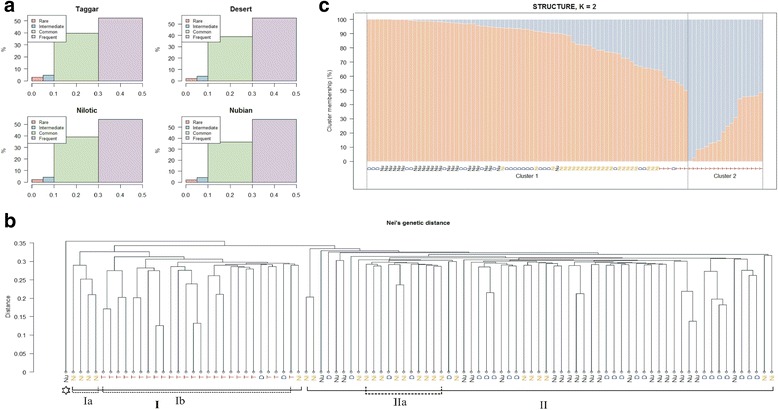
Genetic diversity among Sudanese Goats. Genetic diversity between Taggar (T), Desert (D), Nubian (NU), and Nilotic (NI) goats and minor allele frequency distribution in Sudanese goat breeds. **a** Minor allele frequency distribution of 50 K BeadChip SNP in Sudanese goat breeds. The rare (≥0.0 and ≤0.05), intermediate (≥0.05 and ≤0.1), common variant (≥0.10 and ≤0.3), and the frequent (≥0.3 and ≤0.5) variant MAFs are in green, purple, orange and yellow color, respectively. **b** Hierarchical clustering dendrogram based on Nei’s genetic distance. The Y axis of the dendrogram represents the distance between individuals or clusters of individuals. The X axis represents every individual of the four breeds and shows clusters. One outlier from Nubian goat (*) is placed at much higher distance. **c** STRUCTURE analysis with two clusters (K = 2), each individual is represented by a vertical bar

The percentage of polymorphic SNPs within each breed among all 48,505 SNPs that passed the quality control was very high, ranging from 96.9% (Taggar goat) to 98.2% (Desert goat) (Table 1). After removing SNPs with MAF below 0.05, the average minor allele frequency, was about the same for Nubian (0.31 ± 0.12), Desert (0.31 ± 0.12), Nilotic (0.31 ± 0.13), and Taggar (0.30 ± 0.13) goats. The distribution of minor allele frequencies across the Sudanese goat populations are represented in Fig. 1a. Rare variant MAFs were observed in about 3.1%, 2.2%, 2% and 1.8% in Taggar, Nilotic, Nubian, and Desert goat breeds respectively.

**Table 1:** Diversity indices comparing Sudanese goat breeds

Breed name	Breed acronym	N	H_E_	H_o_	F_IS_	PIC (%)
Nubian	NU	23	0.39	0.39	0.0010	98.0
Desert	D	24	0.39	0.39	0.0041	98.2
Taggar	T	24	0.39	0.39	−0.0129	96.9
Nilotic	NI	24	0.40	0.39	0.0094	97.8
Total		95	0.40	0.39	N.A.	N.A.

N: number of animals; H_E_: expected heterozygosity based on the observed genotype frequencies; H_O_: observed heterozygosity; F_IS_: Wright’s inbreeding coefficient, significance was determined using the diversity package, after 1000 bootstraps the 95% bias corrected confidence interval was used to determine if the estimated F_IS_ is significantly different from 0; PIC: polymorphic information content; N. A.: Not applicable

Additionally, observed and expected heterozygosity values were the same for the examined Nubian, Desert, and Taggar populations (0.39). For Nilotic goats, the expected heterozygosity (0.40) was slightly higher than observed (0.39). The high degree of heterozygosity and the coincidence of expected and observed heterozygosity indicate high genetic diversity and near to zero inbreeding within each breed. This was further confirmed by the inbreeding coefficients (F_IS_). The population inbreeding coefficients (F_IS_) ranged from −0.0129 in Taggar goats to 0.0094 in Nilotic goats and did not significantly differ from zero (Table 1). These values indicate that there is neither inbreeding nor strong kinship within the studied populations. To further confirm this observation, we calculated the kinship matrix using the ‘kinship’ function from the EMMA package [35], EMMA calculates kinship based on identity by state (IBS), meaning that two unrelated individuals measured using SNP markers on average show a kinship coefficient of 0.5, when looking at the values calculated we that kinship between most individuals ranges between 0.64 and 0.71. Between two pairs of individuals we find a slightly increased kinship of 0.81. EMMA analysis confirms that no strong kinship exists between individuals (Additional file 4: Figure S2).

### Genetic diversity between breeds

The estimates for pairwise genetic differentiation between populations (F_st_) were low and varied between 0.0053 (Nubian vs. Desert) and 0.0229 (Nubian vs. Taggar) (Table 2). F_st_ values between Desert, Nilotic and Nubian goats were below 0.0076. The highest genetic distance was found between Taggar and the three other breeds (F_st_ ≥ 0.0134). Only the Taggar breed showed significant genetic differentiation towards the three other breeds. However, pairwise F_st_ values for the other breeds did not significantly deviate from zero. As such F_st_ analysis only provides evidence for population differentiation of Taggar goats versus the other three goat breeds, and no such evidence for population differentiation between the Desert, Nilotic or Nubian. We investigated the SNP by SNP F_st_ values (Additional file 5: Figure S3.) from the supplemental figure it becomes clear that using F_st_ values we are unable to observe regions which explain inter-breed differences. This motivated us to perform additional PCA analysis to further look for other ways to find regions which show inter-breed differences. AMOVA confirmed the high within population variation and low differentiation between populations. The largest proportion of the variation (93.04%) was attributable to the variation within individuals (Additional file 6: Table S2). Differences between populations accounted for only 6.96% of the variance, but was highly significant (*P* < 1.0 * 10^−6^).

**Table 2:** Estimated pairwise fixation indices (F_st_)

Population	Taggar	Desert	Nilotic	Nubian
Desert	**0.0179**	x		
Nilotic	**0.0134**	0.0076	x	
Nubian	**0.0229**	0.0053	0.0072	x
Combined^*^	*0.0032*	−0.0024	−0.0031	−0.0012

^*^Combined: The breed mentioned in the column against the other three breeds combined, bold values indicate significance after 1000 bootstraps, with an alpha level of 0.005 (Bonferonni adjusted multiple testing *p*-value), *cursive values* indicate suggestive significance after 1000 bootstraps, with an alpha level of 0.01

Hierarchical clustering using Nei’s genetic distances clearly shows two different clusters (Cluster I and II) (Fig. 1b). Within Cluster I, the Taggar population forms a distinct group (Cluster I group b); however, we observed two Desert goat individuals positioned within the Taggar cluster. In addition, four Nilotic goat individuals clustered closely together and formed a distinct subgroup (Cluster I group a) which shows a closer relationship to Taggar goats (Cluster I group b). Cluster II comprises all Nubian goats, except one outlier (marked with a * in Fig. 1b), and the remaining Desert and Nilotic goats. Within cluster II, Nilotic goats clustered in a smaller sub-group (Cluster II group a) with an approximately Nei’s genetic distance of 0.31 between individuals belonging to this cluster. None of the Taggar goats was assigned to cluster II. Similar clusters were observed when SNP data was clustered using different distances measurements, such as Manhattan or Reynold distances (Additional file 3: Figure S1).

### STRUCTURE analysis

The genetic structure among all goats was studied using a Bayesian model-based approach that assigns each individual to one or more populations based on the allele frequencies detected at different loci. Following STRUCTURE analysis the posterior probability (Ln *P*(*D*)) of the data indicated the most optimal K value to be 2 (Additional file 7: Table S3).

For K > 2, there is a higher variance of the log likelihood values, while the mean log likelihood of models K > 2 is worse compared to the K = 2 model. With K = 2, individuals were assigned into two main genetic clusters. When looking at a default clustering threshold of 50%, cluster 2 contained 18 out of 24 Taggar goats, while cluster 1 comprised of Nubian, Desert, Nilotic, and six Taggar goats (Fig. 1c). However, when we put the clustering threshold at 60% contribution compared to the default 50% used by STRUCTURE all Taggar goats, and a single Desert goat is in the second cluster.

Results from the STRUCTURE analysis with K = 2 to 5 is summarized in Additional file 7: Table S3, the graphical STRUCTURE plots of the Sudanese goat breeds from *K* = 2 to 5 are shown in Additional file 8: Figure S4.

### PCA analysis

**Fig. 2:**
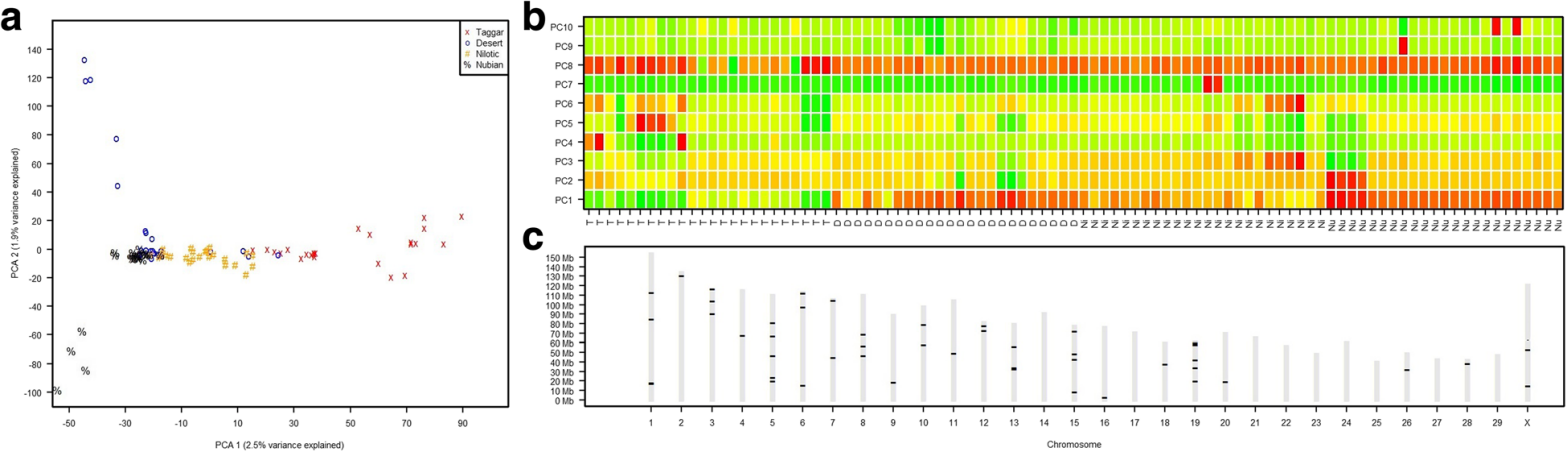
Principal component analysis of all individuals. **a** PC1 (X axis) versus PC2 (Y axis) are shown. Individuals of different breeds are differently colored. PC1 clearly separates between Taggar, Nilotic and a mix between Desert and Nubian goats. The second component allows separating between Desert and Nubian goats. Desert goats are scattered throughout all clusters. The first two principal components, PC1 and PC2, account for 2.5% and 1.9% of the total variance, respectively. **b** Visual representation of the contribution of individuals to the first 10 principal components. The contribution of each individual to each PC1 through 10 is shown using a heat map image. Green color indicates a high contribution to the PC, while yellow shows average contribution, orange is low contribution, and the red color indicates the lowest contribution. **c** Genomic location of SNP contributing to the principle component 1 (PC1) – PC1 allows for the separation of the Taggar, Nubian and Nilotic goat breeds

PCA was used to assess how different the four Sudanese goat populations are. Figure 2a shows the individual loadings of the first and second principal component (PC) against each other. We observed that the loading of PC1 resulted in tight clustering by breed. PC1 could separate three out of four different subpopulations, namely Taggar, Nilotic and a mix of Desert and Nubian goats, this is also seen in Fig. 2b (contribution of individuals to each PC) where the Taggar goats are colored green (high contribution), the Nilotic goats are mostly yellow (average contribution), and the other 2 breeds have a low contribution to the first principal component. Although this separation is not perfect, we assume this could be caused by misclassification of individuals by the owners. We do not observe another principal component in the first 10 components investigated that cause this clear of a separation between breeds, which is expected since PCs are ordered by their variance explained. PC2 was able to separate some of the Nubian (PC2 ≤ 0) from the Desert goat population (PC2 > 0) this is not clearly observable in Fig. 2b, since the classification here seems to depend on 5 Desert goats in green (high contribution) versus 4 Nubian goats in red (low contribution). The first two principal components explained 2.5% (PC1) and 1.9% (PC2) of the genetic variation observed between all individuals. PC1 and PC2 together seemed to be able to almost classify samples into their respective breed, we do observe overlap of some of the Desert goat samples with Taggar, Nilotic and Nubian goats. PCA using 48,505 SNPs allowed us to detect which SNPs contributed highly to PC1 and thereby to the genetic differentiation between Taggar, Nilotic and Nubian goats. To investigate the SNPs which have a high contribution to PC1, we generated a list of SNPs which had a 10 times higher contribution to PC1 compared to the expected contribution (Additional file 9: Sheet 1). We found that 49 SNPs out of 48,505 contributed highly to the PC1, which are visualized in Fig. 2c.

### Relationship between F_st_ and contribution to PC1

**Fig. 3:**
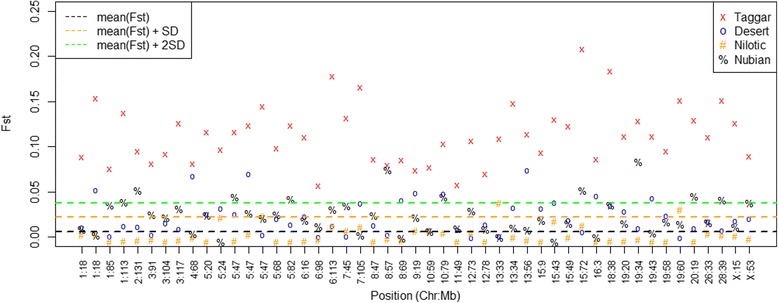
F_st_ values of SNPs contributing to PC1. The F_st_ values of the four different breeds against the combination of the three other goat breeds at the positions of the SNPs contributing to PC1. The SNPs selected by PCA analysis, show F_st_ values higher than at least mean(F_st_) + 2 SDs

To further investigate our results that SNPs contributing highly to PC1 are biologically important in the distinction between Taggar goats versus the other Sudanese breeds. We explored this by investigating if these SNPs show higher than average differentiation. We used the F_st_ values of the SNPs which contributed highly to PC1. Since this would give us an independent line of evidence that these SNPs are important in the differentiation of the Taggar goat versus the other goat breeds, and that our PCA approach allows us to identify these regions. We therefore plot the F_st_ values of the four different breeds against the combination of the three other breeds at the positions of the SNPs contributing to PC1 (as can been seen in Fig. 3). We observe significant higher F_st_ values in the Taggar versus the other goat breeds at these SNPs. All 49 SNPs selected by PCA analysis, show F_st_ values higher than at least mean(F_st_) + 2 SDs. This means that these SNPs show signs of differentiation in Taggar goats versus the other breeds. This is not observed for any of the other three goat breeds. Desert goats versus the other breeds showed only 5 SNPs above threshold, Nilotic goats versus the other breeds showed no SNPs above the threshold, and Nubian versus the rest showed 8 SNPs above the threshold, this means that the regions contributing to PC1 are regions of high differentiation in Taggar goats, but are not regions of high differentiation in the other Sudanese breeds.

### Genes near SNPs contributing to PC1

To investigate regions which show a high contribution to PC1 and have a high F_st_ values in Taggar versus the other goats, we selected genes from a region of 500 kb around those SNPs. We extracted 208 genes (Additional file 9: Sheet 2). Among these genes we found genes known to be involved in the following physiological traits:

- Bone formation (*sclerostin, epiphycan, bone morphogenetic protein receptor type-1A precursor, arylsulfatase G, mesenchyme homebox 1, ClpB homolog mitochondrial AAA ATPase chaperonin*)
- Blood (water balance, glucose, and salts) homeostasis (*vasopressin, haptoglobin, hepatocyte growth factor activator*), sodium/potassium/calcium exchanger (solute carrier family 24 member 5)
- Heart and muscle (*phospholamban*, myosin phosphatase, mitochondrial cardiolipin hydrolase)
- Growth / dwarfism (*Stanniocalcin-1, inhibitor of growth protein 5*)
- Eye development (*keratocan, lumican, melanopsin, epiphycan, retinal guanylyl cyclase 2, beta-crystallin A3, all-trans-retinol 13,14-reductase*)
- Coat color (*sodium/potassium/calcium exchanger 5*)

Unfortunately, gene-ontology (GO) is not available for goats, so we are unable to perform GO testing on these groups of genes. Their location around the SNPs that contributed highly to PC1 and the high F_st_ values observed in Taggar goats makes these genes together with the other positional candidate genes interesting targets for the analysis of adaptation found between the different Sudanese goat breeds.

## Discussion

This study was conducted to contribute to the genetic characterization of the economically most interesting indigenous goat breeds in Sudan. Using the 50 k goat SNP chip, we examined the genetic diversity within and between breeds, the breed differentiation, and the genetics contributing to breed differences.

High levels of genetic diversity within the examined goat populations were observed. More than 96% of SNPs that passed the quality check were polymorphic in each breed albeit SNPs on the chip were selected from breeds such as Saanen, Alpine, Creole, Boer, Kacang, and Savanna and did not consider indigenous East African breeds [19]. Since the expected and observed heterozygosity in the examined Sudanese goat breeds were similar (0.39), we did not find heterozygote deficiency. Using the same SNP chip, heterozygosity in Sudanese goat breeds was similar to Bakri goats in Egypt (0.40) [36], Ethiopian goats breeds (0.38) [37] and Angora goats from South Africa (0.37) [38]. Consistent with low heterozygote deficiency and high allelic diversity, inbreeding within Sudanese goat breeds was also close to zero. The inbreeding coefficient estimated for Sudanese Nubian goats (F_IS_ = 0.001) was consistent with previous findings for Nubian goats in Ethiopia (F_IS_ = 0.073) [37]. This outcome indicates the highly diverse genetic reservoir of Sudanese goats.

The low F_st_ values among Sudanese breeds indicate low genetic differentiation among these populations, which in turn mirrors the population history with a likely common origin and the recent husbandry system in connection with nomadic traditions. AMOVA further supported this finding by providing evidence that most variation was distributed within individuals and to a lesser extent genetic variation (6.96%) was explained by differences among the Sudanese goat populations.

By applying different genetic distance clustering methods, STRUCTURE and PCA, we observed clear separation of Taggar goats towards the other goat breeds. Taggar, which is a dwarf goat, and was identified as the most genetically distinct group in respect to other goat populations in this study. The output of the STRUCTURE analysis at K = 2 further supported this finding. It clearly distinguished Taggar from the other Sudanese goat breeds. This finding could be explained by the fact that Taggar goats are geographically isolated in the mountain regions of Sudan. Mountain regions could have caused natural selection of small animals which are more nimble and feed efficient, which over the course of many generations could have led to the dwarfism phenotype we observe currently in Taggar goats. This natural selection might have caused genetic signatures in Taggar not observed in the other three breeds. Additionally, since Taggar goats are geographically separated (mountain versus low-lands) this might have played a limiting role in their ability to mate with the other three low-land goat breeds in Sudan, thus explaining why they show a clear separation from the other goat breeds.

It is observed that Desert goats are most scattered amongst the other two goats (Nilotic and Nubian). This could be due to Desert goat husbandry: (1) forced by changing market conditions, they began to shift from dual purpose to dairy goats by crossing their Desert goats with Nubian goats [39], and (2) Desert goats are owned by nomadic tribes who use communal grazing lands and watering points, where different herds meet and randomly mate which leads to gene flow.

Based on Nei’s genetic distance we find a closer relationship between Taggar and a sub-group of Nilotic goats, which could be attributed to the low geographical distance between the two populations in the mountain area of the Southern part of Sudan.

Principal component analysis showed that the first two principal components could be used to differentiate between Sudanese goat breeds and to assign individuals to a particular breed. PC1 shows separation of Taggar goats, Nilotic goats, and a mixed of Desert and Nubian goats. PC2 seems to be able to differentiate between Nubian and Desert goats, though some misclassifications still remain. SNPs contributing highly to PC1 allowed us to define regions of the genome at which the Taggar goats are significantly different from the other three breeds. Among the genes in the vicinity of the SNPs contributing to PC1, we identified genes which might be interesting candidates when looking at breed characteristics and adaptation differences between Sudanese breeds studied here. Among them we found genes known to contribute to bone formation, blood homeostasis, heart and muscle development, growth, eye development, and coat color.

Within the list of the genes there are genes such as *sclerostin* and *bone morphogenetic protein receptor type-1A precursor* known to have an effect on the bone morphology and formation. Studying differences in these genes could elucidate the underlying genetics of the differences between Sudanese goat breeds in regard to bone formation and body measurements characteristics. The *Stanniocalcin-1* gene on chromosome 8 is one of the genes, which is interesting for the discrimination among breeds, since Taggar goats are a species of achondroplastic dwarf goats with lack of ossification at the cartilage joints [13, 14]. *Stanniocalcin-1* acts as a paracrine regulator of growth plate chondrogenesis [40]. This gene is known to cause dwarfism in mice when it is over expressed [41].

Several other genes that are important for blood metabolism and homeostasis fell within regions in the PC1. This might reflect the adaptation of the Sudanese goat breeds to different environmental conditions. An example is the Desert goat which can survive in the areas where the lack of water resources is dominant. In a similar way, we detected candidate genes in regions which differ between the goat breeds for heart and muscle characteristics. Since natural variation is present between the breeds differences in these genes could provide targets for genomic breeding to improve meat quality.

The detection of genomic differences based on the principle component 1 (PC1) which allows for the separation of the Sudanese goat breeds can shed light on the genes underlying the adaptations. Natural variation present between these breeds provide a unique opportunity to improve local breeds through breeding using genomic markers or to breed improved resistance to harsh climates into imported high production breeds. Therefore, further research is required to identify the genomic regions which are associated with different important economical traits in Sudanese goat breeds.

## Conclusions

Based on our genome-wide analysis of SNPs in Sudanese goats, this study shows that Taggar goats show significant genetic differences from the other three breeds studied. We further conclude that the first principal components allow us to differentiate between Sudanese goat breeds. Furthermore F_st_ values of these SNPs show high differentiation for Taggar goats, but no significant differentiation for the other breeds under study. Genes in the proximity of these SNPs contributing highly to the first principal component might be interesting candidates when looking at breed characteristics and adaptation differences in Taggar goats.

## Additional files

Additional file 1: Table S1. Sample locations. (DOC 44 kb)

Additional file 2: Locations of protein coding genes. (TXT 3463 kb)

Additional file 3: Figure S1. Reynolds and Manhattan distances. (PNG 40 kb)

Additional file 4: Figure S2. Kinship. (PNG 71 kb)

Additional file 5: Figure S3. F_st_ (PNG 52 kb)

Additional file 6: Table S2. AMOVA. (DOCX 11 kb)

Additional file 7: Table S3. STRUCTURE. (DOC 33 kb)

Additional file 8: Figure S4. STRUCTURE analysis of Sudanese goat breeds. (PNG 102 kb)

Additional file 9: SNPs and genes contributed to PCA. (XLSX 36 kb)

Additional file 10: Raw genotype data obtained from the Goat SNP52 BeadChip. (XLSX 18918 kb)

## Abbreviations

AMOVA: Analysis of molecular variance
BLAST: Basic Local Alignment Search Tool
GO: Gene-ontology
He: Heterozygosity expected
Ho: Heterozygosity observed
HWE: Hardy–Weinberg equilibrium
IBS: Identity by state
IGGC: International Goat Genome Consortium
MAF: Minor Allele Frequency
PC: Principal Component
PCA: Principal Component analysis
SD: Standard deviation
SNAP: SNP Annotation and Proxy Search
SNP: Single Nucleotide Polymorphism
