# Supplementary material for: Whole genome population genetics analysis of Sudanese goats identifies regions harboring genes associated with major traits
Source: BMC Genet. 2017 Oct 23;18:92. doi: 10.1186/s12863-017-0553-z (PMC5651574; doi:10.1186/s12863-017-0553-z)
Supplement: Supplementary file 1 — Sample locations. (DOC 44 kb) [file 12863_2017_553_MOESM1_ESM.doc]

| **Breed** | | **Sample No** | **Location** | **Source** | **State** |  |
| --- | --- | --- | --- | --- | --- | --- |
| Nubian | | 4 | Aldamir | University farm | River Nile |  |
|  |  | 3 | Aljazirah | Village (3) | Aljazirah |  |
|  |  | 1 | Aljazirah | Research station | Aljazirah |  |
|  |  | 4 | Shendi | Village (4) | River Nile |  |
|  |  | 4 | Khartoum | Districts (3) | Khartoum |  |
|  |  | 4 | Abu Hamad | Research station | River Nile |  |
|  |  | 3 | Dongola | Village (3) | Northern |  |
|  |  | 1 | Dongola | Research station | Northern |  |
| Desert | | 20 | Bara | Village (8) | North Kordofan |  |
|  |  | 4 | Abu Zabad | University farm | North Kordofan |  |
| Taggar | | 19 | Dalang area | Village (5) | South Kordofan |  |
|  |  | 5 | Nuba Mountains | Research station | South Kordofan |  |
| Nilotic | | 21 | Kosti | Village (7) | White Nile |  |
|  |  | 3 | Rabak | Village (1) | White Nile |  |
|  |  | | | | | |

**Table S1: Sample location**
