# Supplementary figures and images for: Whole genome population genetics analysis of Sudanese goats identifies regions harboring genes associated with major traits
Source: BMC Genet. 2017 Oct 23;18:92. doi: 10.1186/s12863-017-0553-z (PMC5651574; doi:10.1186/s12863-017-0553-z)

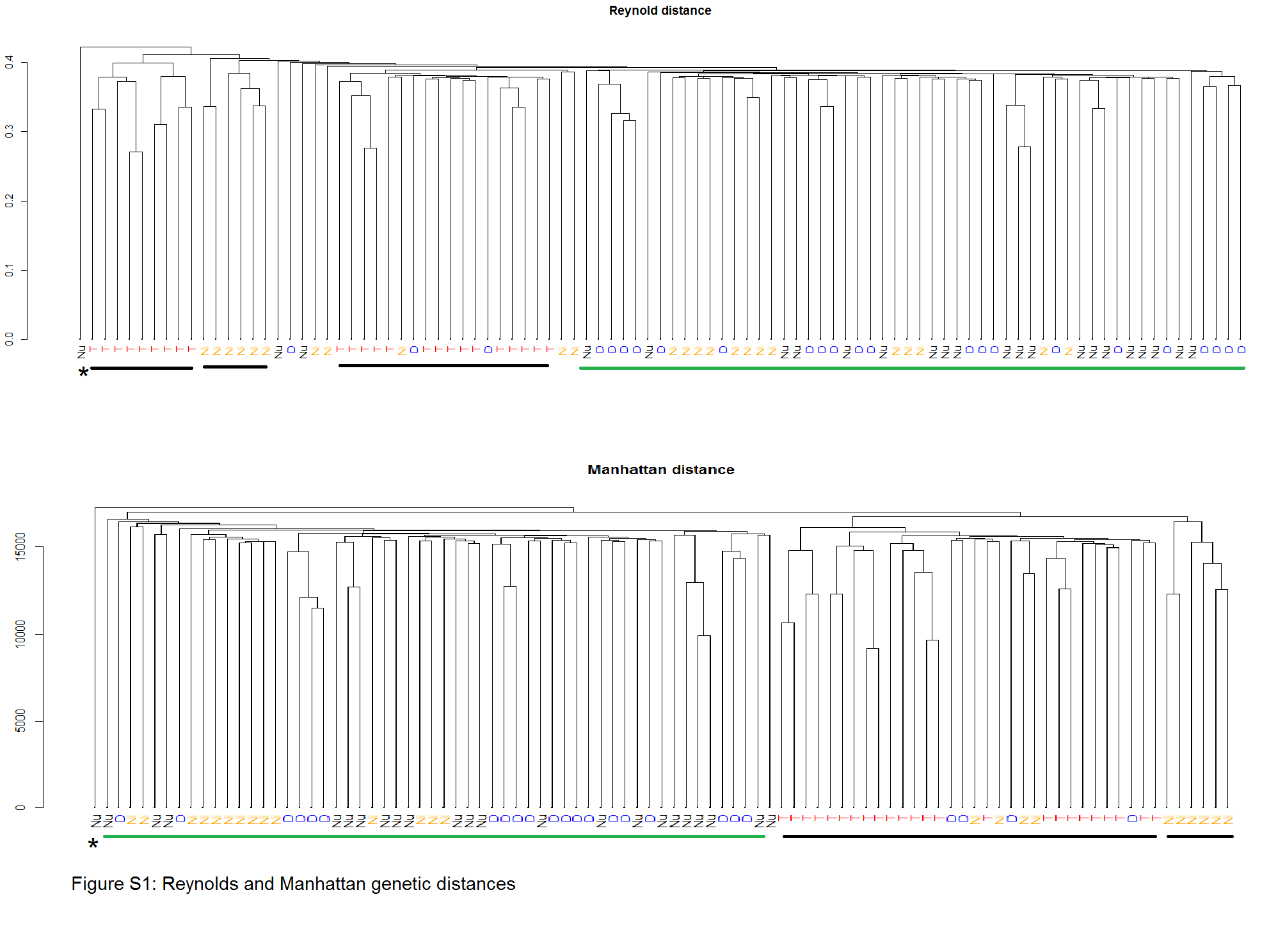

Supplement: Supplementary file 3 — Reynolds and Manhattan distances. (PNG 40 kb) [file 12863_2017_553_MOESM3_ESM.png]

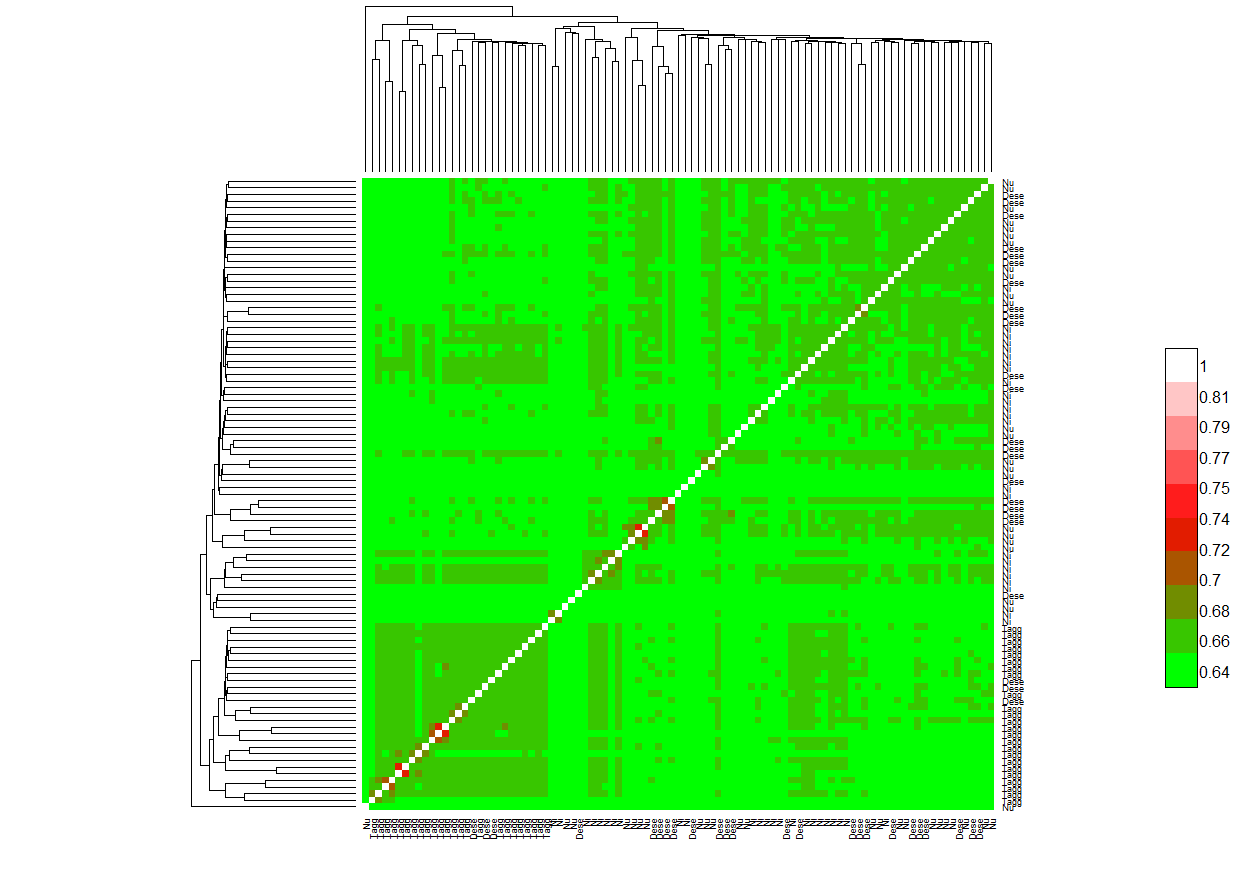

Supplement: Supplementary file 4 — Kinship. (PNG 71 kb) [file 12863_2017_553_MOESM4_ESM.png]

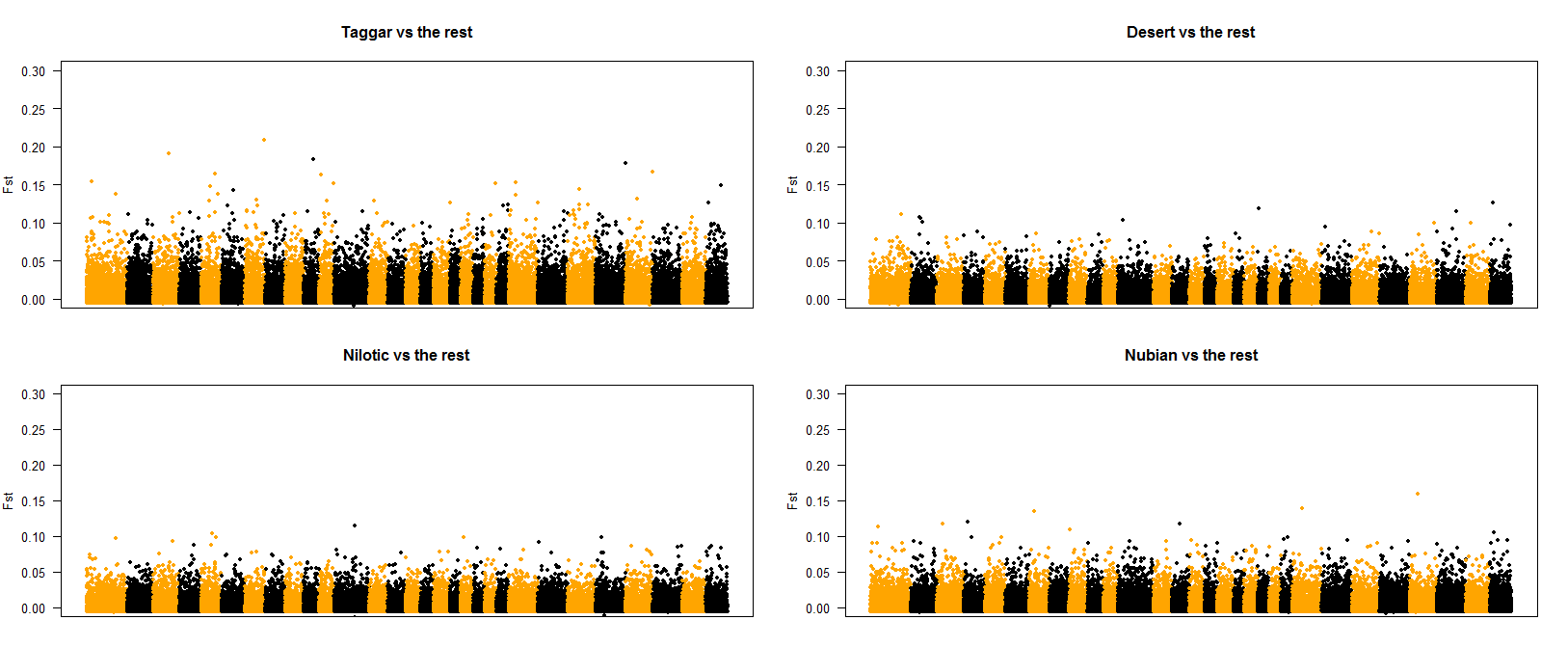

Supplement: Supplementary file 5 — Fst (PNG 52 kb) [file 12863_2017_553_MOESM5_ESM.png]

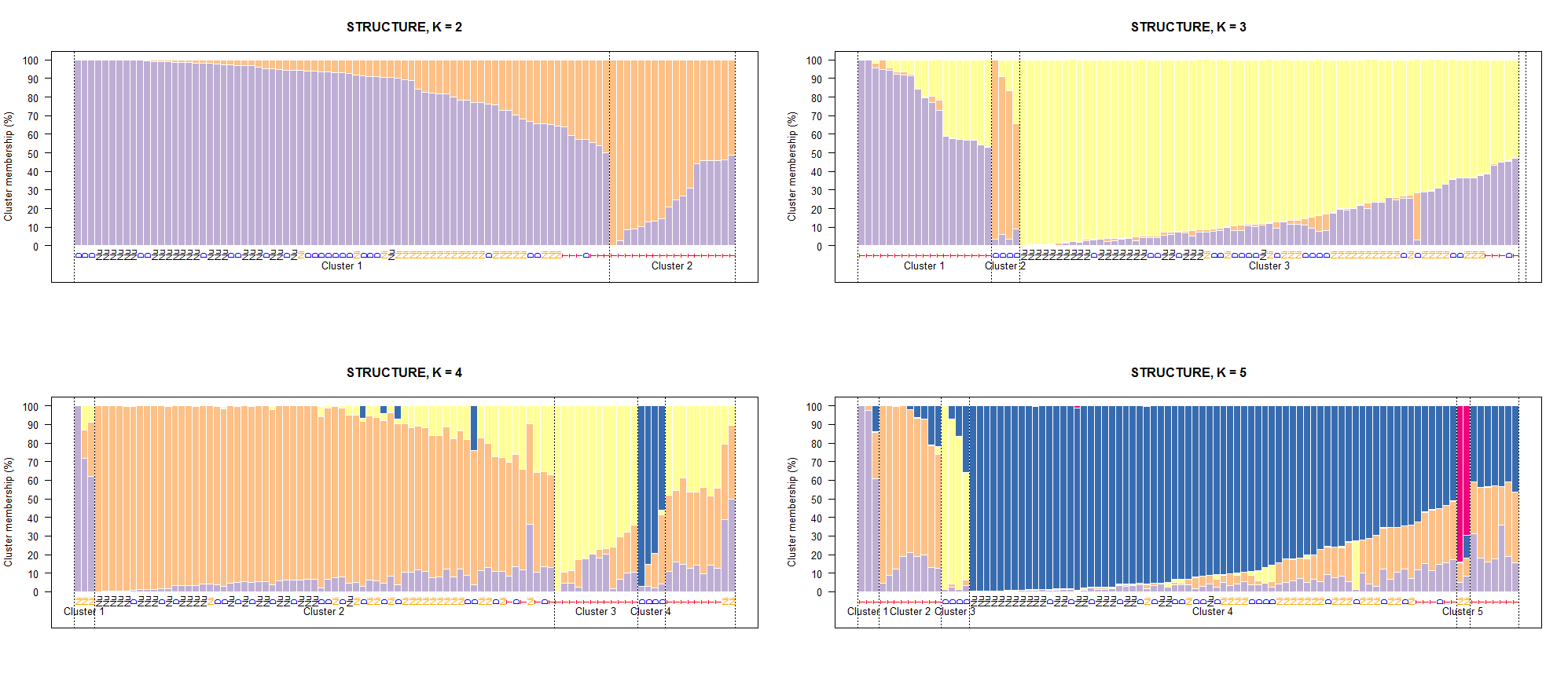

Supplement: Supplementary file 8 — STRUCTURE analysis of Sudanese goat breeds. (PNG 102 kb) [file 12863_2017_553_MOESM8_ESM.png]
