# Supplementary material for: Whole genome population genetics analysis of Sudanese goats identifies regions harboring genes associated with major traits
Source: BMC Genet. 2017 Oct 23;18:92. doi: 10.1186/s12863-017-0553-z (PMC5651574; doi:10.1186/s12863-017-0553-z)
Supplement: Supplementary file 6 — AMOVA. (DOCX 11 kb) [file 12863_2017_553_MOESM6_ESM.docx]

**Table S2: Analysis of molecular variance (AMOVA) in the four goat populations**

| Source of variation | d.f. | SSD | MSD | Variance components | Percentage of  variation |
| --- | --- | --- | --- | --- | --- |
| Among populations | 3 | 0.296 | 0.099 | 0.002 | 6.96 |
| Within individuals | 91 | 3.956 | 0.044 | 0.043 | 93.04% |
| Total | 94 | 4.252 | 0.045 |  | 100 |
| d.f.: Degree of freedom; SSD: Sum of squares; MSD: Mean squared deviations | | | | | |
