# Supplementary material for: Whole genome population genetics analysis of Sudanese goats identifies regions harboring genes associated with major traits
Source: BMC Genet. 2017 Oct 23;18:92. doi: 10.1186/s12863-017-0553-z (PMC5651574; doi:10.1186/s12863-017-0553-z)
Supplement: Supplementary file 7 — STRUCTURE. (DOC 33 kb) [file 12863_2017_553_MOESM7_ESM.doc]

**Table S3.** F_st_, Likelihood of K from STRUCTURE and mean value of alpha

|  | K=2 | K=3 | K=4 | K=5 |
| --- | --- | --- | --- | --- |
| F_ST_ 1 | 0,0041 | 0,084 | 0,182 | 0,2092 |
| F_ST_ 2 | 0,0901 | 0,1785 | 0,0047 | 0,113 |
| F_ST_ 3 |  | 0,0043 | 0,0967 | 0,1661 |
| F_ST_ 4 |  |  | 0,1764 | 0,0045 |
| F_ST_ 5 |  |  |  | 0,2753 |
|  |  |  |  |  |
| Estimated Ln probability of data | -5417697 | -5408406 | -5403729 | -5398950 |
| Mean value of ln likelihood | -5392519 | -5370226 | -5351220 | -5332560 |
| Variance of ln likelihood | 50356,9 | 76359,3 | 105018,2 | 132779,3 |
| Mean value of alpha | 0,3135 | 0,2239 | 0,0921 | 0,0826 |
